# Prolactin and Growth Hormone Signaling and Interlink Focused on the Mammosomatotroph Paradigm: A Comprehensive Review of the Literature

**DOI:** 10.3390/ijms241814002

**Published:** 2023-09-12

**Authors:** Marta Araujo-Castro, Mónica Marazuela, Manel Puig-Domingo, Betina Biagetti

**Affiliations:** 1 Department of Endocrinology and Nutrition, Hospital Universitario Ramón y Cajal, Colmenar Viejo Street km 9, 28034 Madrid, Spain; 2 Instituto de Investigación Biomédica Ramón y Cajal (IRYCIS), Colmenar Viejo Street km 9, 28034 Madrid, Spain; 3 Department of Endocrinology and Nutrition, Hospital Universitario La Princesa, 28006 Madrid, Spain; 4 Centro de Investigación Biomédica en Red de Enfermedades Raras (CIBERER GCV14/ER/12), Monforte de Lemos Avenue, 28029 Madrid, Spain; 5 Department of Endocrinology and Nutrition, Department of Medicine, Germans Trias i Pujol Research Institute and Hospital, Universitat Autònoma de Barcelona, 08916 Badalona, Spain; 6 Centro de Investigación Biomédica en Red de Enfermedades Raras CIBERER G747, Monforte de Lemos Avenue, 28029 Madrid, Spain; 7 Department of Endocrinology and Nutrition, Vall d’Hebron University Hospital, Reference Networks (ERN) and Vall d’Hebron Research Institute (VHIR), Vall d’Hebron Avenue, 119, 08035 Barcelona, Spain; 8 Diabetes and Metabolism Research Unit, Vall d’Hebron Research Institute and CIBERDEM (ISCIII), Universidad Autónoma de Barcelona, Avenida Can Domènech s/n, 08193 Bellaterra, Spain

**Keywords:** acromegaly, prolactin, pituitary neuroendocrine tumors, growth hormone, mammosomatotroph, prolactin receptor, dopamine receptor, somatostatin receptor, plurihormonal tumors

## Abstract

Prolactin (PRL) and growth hormone (GH) are peptide hormones that bind to the class 1 cytokine receptor superfamily, a highly conserved cell surface class of receptors. Both hormones control their own secretion via a negative autocrine loop in their own mammosomatotroph, lactotroph or somatotroph. In this regard, GH and PRL are regulated by similar signaling pathways involving cell growth and hormone secretion. Thus, GH and PRL dysregulation and pituitary neuroendocrine tumor (PitNET) development may have common pathogenic pathways. Based on cell linage, lactotroph and somatotroph PitNETs come from pituitary-specific POU-class homeodomain transcription factor (Pit-1). Mammosomatotroph and plurihormonal PitNETs are a unique subtype of PitNETs that arise from a single-cell population of Pit-1 lineage. In contrast, mixed somatotroph–lactotroph PitNETs are composed of two distinct cell populations: somatotrophs and lactotrophs. Morphologic features that distinguish indolent PitNETs from locally aggressive ones are still unidentified, and no single prognostic parameter can predict tumor aggressiveness or treatment response. In this review, we aim to explore the latest research on lactotroph and somatotroph PitNETs, the molecular mechanisms involved in PRL and GH axis regulation and the signaling pathways involved in their aggressiveness, particularly focused on mammosomatotroph and mixed subtypes. Finally, we summarize epidemiological, clinical, and radiological features of these exceptional tumors. We aim to shed light, from basic to clinical settings, on new perspectives and scientific gaps in this field.

## 1. Introduction

Prolactin (PRL) and growth hormone (GH) represent groups of hormones that share similar chemical structures [1,2]. Both hormones are regulated by similar signaling pathways involving cell growth and hormone secretion. Thus, GH and PRL dysregulation and pituitary tumor development may have common pathogenic pathways.

Pituitary neuroendocrine tumors (PitNETs) are a group of tumors that arise from the pituitary gland [3]. Particularly, based on cell linage, lactotroph and somatotroph tumors come from pituitary-specific POU-class homeodomain transcription factor (PIT-1). A recent study estimates that up to 30% of patients with acromegaly have mammosomatotroph tumors and 13% have mixed somatotroph–lactotroph tumors based on immunohistochemistry [4].

Transsphenoidal surgery is, in general, the first-line treatment selected for patients with secretory PitNETs, independently of size, except for lactotroph tumors [5], for which dopamine agonists (DA), mainly cabergoline, are the first choice. However, these tumors are frequently resistant to DA [6,7,8,9,10] and tend to be more aggressive in young males [9,10,11]. Likewise, they are more refractory to treatment than somatotroph tumors [12,13,14]. 

Overall, these facts reveal that clinical and biochemical presentation, behavior and response to treatment of these tumors can be considered highly heterogeneous. Therefore, better understanding of the molecular pathways involved in these tumors’ development is essential for identifying patients harboring aggressive lesions and establishing personalized therapeutic options.

In this review paper, we aim to explore the molecular mechanisms involved in PRL and GH axis regulation. We also discuss the latest research on lactotroph and somatotroph PitNETs, as well as the signaling pathways involved in their aggressiveness, particularly focused on mammosomatotroph and mixed tumors. The review concentrates on the central effects of GH and PRL, omitting any discussion of their peripheral actions. We also summarize epidemiological, clinical and radiological features of these exceptional tumors. We aim to shed light, from basic to clinical settings, on new perspectives and scientific gaps in this field.

## 2. Methods

This narrative review was conducted following the SANRA scale [15]. The search strategy was conducted in PubMed, without a date filter, up to the end of June 2023. The search terms that we used are described in Table 1. Two independent reviewers (BB and MAC) chose the potentially relevant articles retrieved after reading the title, abstract or whole article and discarded repeated articles. Only articles published in English were included. The articles identified by these searches and relevant references cited in those articles were reviewed. We largely selected articles published in the past 20 years but did not exclude seminal older articles. After this, 112 papers were included.

## 3. Ontogeny and Cell Linage

**Figure 1:**
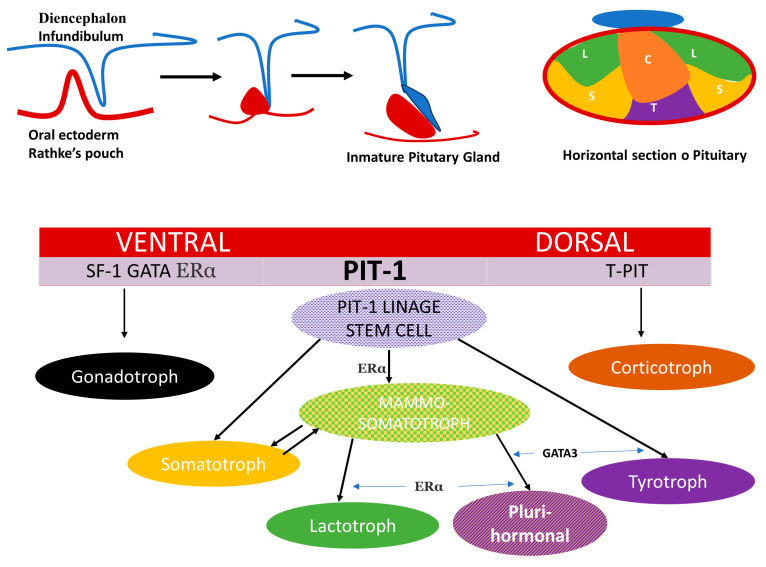
Schematic representation of pituitary development, distribution of cell subtypes in the anterior lobe and spatial activation of transcription factors. The composite structure of the adult pituitary has a dual embryonic origin. The posterior lobe consists of nervous tissue arising from the diencephalon and represents an extension of the hypothalamus and the anterior lobe derives from the oral ectoderm. The dorsal and ventral side of the embryonic pituitary generate proliferative and positional signals which regulate the expression of transcription factors. In the ventral side when SF1, GATA, ERα are activated and determine the gonadotroph linage. T-Pit signal differentiates the most dorsal cells into corticotroph (C)) (in orange) and Pit-1 induced in the caudomedial region of the pituitary gland, is the most complex with 5 monomorph cell types somatotroph (S) (in yellow), lactotroph (L) (in green), thyrotroph cells (T) (in violet), mammosomatotrophs (in yellow mixed texture) and Plurihormonal linage tumor(in violet mixed texture).

Pituitary organogenesis begins during week 4 of fetal development with the formation of the hypophyseal placode, which gives rise to Rathke’s pouch. At the same time, a downward extension of the ventral diencephalon forms the posterior lobe; the two lobes connect to form the composite structure of the adult pituitary [16]. The dorsal and ventral side of the embryonic pituitary generate proliferative and positional signals which regulate the expression of transcription factors [17]. These transcription factors regulate the specific differentiation of the distinct cell type. Ontogeny and cell lineage of PRL and GH cells in the pituitary gland have been studied using animal models and in vitro experiments [18,19]. Mammosomatotrophs, somatotrophs, lactotrophs and thyrotropes arise from Pit-1 expression induced in the caudomedial region of the anterior lobe in the pituitary gland (Figure 1). 

Mammosomatotroph tumors are a unique subtype that arises from a single cell population of Pit-1 lineage that produces both GH and PRL and are also positive for the alpha-subunit [3,20]; some of them, as a consequence of the shared Pit-1 origin, may synthesize and secrete TSH and also express the transcription factor GATA. In contrast, mixed somatotroph–lactotroph tumors are composed of two distinct cell populations: somatotrophs and lactotrophs. These tumors express Pit-1 in all tumor cells, but only the cells that express PRL also express Erα [3,21].

## 4. Prolactin and GH Receptor in the Mammosomatotroph Cell

**Figure 2:**
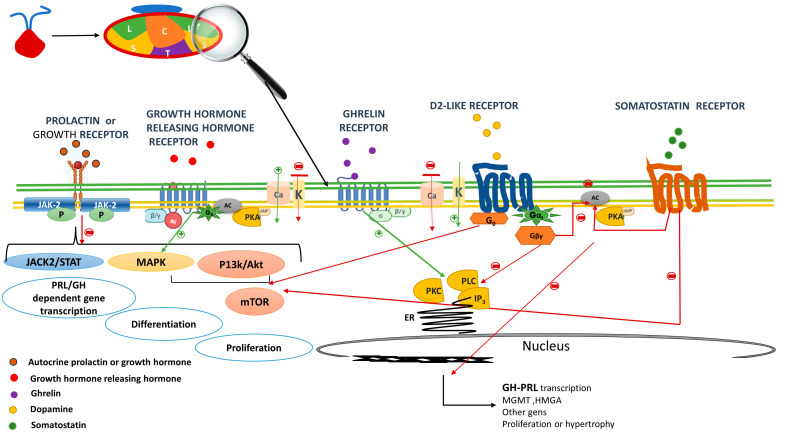
Cell receptors in the mammosomatotroph. The autocrine released GH and PRL bind to prolactin or growth hormone receptor and via Janus kinase-2-signal transducer and activator of transcription-5 (JAK2-STAT5), (PI3K-Akt-mTOR) or the MAPK pathways, mediates changes in transcription and differentiation preventing hormone formation and cell proliferation (negative short loop). Growth hormone releasing hormone receptor (GHRHR) activation results in the secretion and production of growth hormone through cyclic adenosine monophosphate (cAMP)-dependent pathways. The GHRHR activation induces adenylyl cyclase (AC) that generates the conversion of ATP to cAMP; stimulating protein kinase A (PKA) regulatory subunits which increase intracellular Ca^2+^ via voltage-gated Ca^2+^ currents, thus favoring growth hormone secretion. GHRHR also activates MAPK pathways, thus, is related to cellular growth. The downstream signals of the Growth hormone secretagogue receptor includes among other the phospholipase C (PLC)—inositol triphosphates (IP3) pathways. IP3 is soluble and diffuses through the cell, where it binds to its receptor, which is a calcium channel located in the endoplasmic reticulum. When IP3 binds its receptor, calcium is released into the cytosol, thereby activating various calcium regulated intracellular signals potentiating GH but also prolactin secretion. After dopamine binding to Dopamine receptor type 2, K^+^ channels are activated leading to reduce calcium influx, which result in an immediate suppression of prolactin and or GH release. Further decreases in intracellular calcium are achieved by inhibition of PLC and PKC. The main mechanism for the suppression of PRL or GH gene expression is through ACactivity inactivation, resulting in the suppression of PRL or GH gene expression, cell proliferation and decreases the cell size. D2 via G0 also activates phosphatidylinositol 3-kinase (PI3K), and mitogen activated protein kinase (MAPK) pathways to prevent cell proliferation. Similar to D2R, somatostatin receptor (SSTR) inhibits the secretion/synthesis of GH/ PRL mainly through AC inhibition lowering AMPc levels and decreasing intracellular Ca^2+^ concentration via activation of K^+^ channels and the inhibition of voltage dependent Ca^2+^ channels. The anti-proliferative effects of SSTR are mediated among others via PI3K/Akt impairing cell proliferation. Red arrows indicate inhibition a green activation. GH: growth hormone, PRL: prolactin, JAK/STAT: Janus kinase/signal transducer and activator of transcription, PI3K: phosphatidylinositol 3-kinase; ER: endoplasmic reticulum.

PRL and GH are peptide hormones that bind to the class 1 cytokine receptor superfamily, which is a highly conserved cell surface class of receptors [22,23]. Both hormones control their own secretion using a negative autocrine loop in their own mammosomatotroph, lactotroph or somatotroph [24]. Cytokine receptors lack intrinsic protein tyrosine kinase (PTK) activity and, therefore, the activation of signaling pathways requires binding to cytoplasmic PTKs for their signal transduction. These latter categories include the so-called Janus kinase-2-signal transducer and activator of transcription-5 (JAK2-STAT) [25,26], phosphoinositide 3-kinase-Akt (PI3K-Akt-mTOR) or the MAPK pathways to mediate changes in transcription, differentiation and proliferation [27] (Figure 2). The final action observed depends on the target cell and the specific downstream pathway activated. In this review, we focus on the central inhibitory effects of PRL and GH on their own cell proliferation and hormone secretion.

The JAK2/STAT pathway plays a crucial role in various biological processes such as proliferation, differentiation, apoptosis, survival and migration. The inhibitory effects of paracrine/autocrine PRLR/JAK2/STAT5 pathway activation in the lactotroph cell are opposed to the classical proliferative effects of PRL in most other tissues [28], such as the breast, prostate and beta cells. The anti-proliferative effect of PRL in the pituitary are mainly related to the constitutive activation of JAK2/STAT5 in lactotrophs [29,30]. Likewise, Atiprimod treatment in GH3 cells—a model of functional mammosomatotroph tumors—decreased their viability while inhibiting cell growth and colony formation by blocking STAT3 activation in a dose-dependent manner [31]. Thus, alterations in the JAK2/STAT pathway on somatommamotroph cells may contribute to the pathogenesis—and, eventually, to the aggressiveness—of these tumors.

The mTOR pathway regulates the cell cycle and its overactivity has been associated with several cancers [32], as well as with aggressive pituitary tumors [33,34]. The PRL receptor (PRL-R) sequence of 46 PRL-secreting PitNETs found a PRLR variant with gain-of-function in this pathway, which was reverted with everolimus, an mTOR inhibitor [35,36]. Similarly, in GH-secreting PitNETs tissues, knockdown of p300—a histone acetyltransferase coactivator which regulates the transcription of several genes crucial in pituitary tumorigenesis—inhibited cell proliferation and clone formation via mTOR signaling pathways [37]. Thus, mTOR inhibitors could be a promising therapy for PRL- and GH-secreting PitNETs, including mammosomatotrophs.

The MAPK pathway promotes cellular overgrowth-activating proliferative genes and, at the same time, enables cells to overcome metabolic stress by inhibiting AMPK signaling, a key sensor of cell energetic status [38]. Long-term activation of the Ras/MAPK pathway was found to promote differentiation of the bihormonal somato-lactotrope GH4 precursor cell into a prolactin-secreting cell (lactotroph cell phenotype), both in vitro and in vivo [39]. Thus, this pathway could be involved in promoting mamosomatotroph differentiation.

However, in human somatotroph PitNETs, there is a lesser degree of GH receptor (GH-R) expression compared to normal somatotrophs [40]. The action of pegvisomant, an antagonist GH-R, was studied via mRNA expression levels and immunocytochemistry staining of GH-R in 31 pure and mixed GH-/PRL-secreting PitNETs. Pegvisomant induced a dose-dependent inhibition of GH and PRL secretion without impacting cell proliferation [41]. Polymorphisms in GH-R, such as d3-GHR, have been studied in acromegaly, but correlation with clinical features or therapeutic outcomes has not been consistent [34]. Likewise, a variant in GH-R was present in 6/14 sparsely granulated and in 0/12 densely granulated somatotrophs. This GH-R variant was associated with altered GH binding and downstream signaling of the GH-R [42]. Thus, the ineffective sensing of ambient GH and the lack of negative feedback on GH-R could potentially stimulate the tumor growth.

## 5. Dopamine Receptor 

Physiologic PRL secretion occurs mainly under inhibitory stimuli via tuberoinfundibular dopamine (TIDA) neurons in the hypothalamus binding to Type 2 Dopamine Receptor (DRD2) [43]. PRL also controls its own secretion through a short loop negative feedback, stimulating TIDA cells [44] and, as we mention above, in their own mammosomatotroph or lactotroph cell by a negative autocrine loop [24]. 

Dopamine receptors are a class of G protein-coupled receptors. They are grouped into D1-like receptors, including DRD1, DRD5 and D2-like receptors, including DRD2, DRD3 and DRD4. Specifically, DRD2 receptors in the lactotroph cells inhibit PRL secretion and lactotroph proliferation [45].

The activation of DRD2 results in a reduction of PRL exocytosis and gene expression by a variety of intracellular signaling mechanisms. On the one hand, controlling calcium fluxes via K^+^ channels activation leads to membrane hyperpolarization and the inactivation of voltage-gated calcium channels, resulting in the inhibition of PRL release from secretory granules. On the other hand, adenylyl cyclase activity is inactivated, resulting in the suppression of PRL gene expression, inhibiting lactotroph proliferation. Moreover, DRD2 via G0 also activates phosphatidylinositol 3-kinase (PI3K) and mitogen-activated protein kinase (MAPK) pathways, preventing lactotroph proliferation [46] (Figure 2).

Therefore, PRL secretion and lactotroph proliferation pathways are intimately related, which explains in part why, in general (but not invariably), PRL secretion and tumor volume run in parallel in lactotroph PitNETs, as occurs for DA response.

It is remarkable that DRD2 plays a critical role in establishing the specific differentiation ratio between lactotroph and somatotroph cells types from their common mammosomatotroph precursor [47].

Likewise, there is relative high expression of DRD2 not only in PRL-secreting but also in somatotroph PitNETs [48,49]. DAs are the first line treatment for PRL-secreting PitNETs but they are also recommended in the treatment of some patients with acromegaly [50,51]. Thus, DRD2 pathway disruption could cause lactotroph, somatotroph, mammosomatotroph and/or mixed tumor development. In this regard, Friedman et al. [52] studied the presence of inactivated variants of DRD2 gene as a possible link between functional dopamine uncoupling lactotrophs from the inhibitory effects of dopamine and the development of PRL-secreting PitNETs. They used direct DNA sequencing in 79 humans’ pituitary tumors, mostly lactotroph and mixed GH-/PRL-secreting tumors. No mutations were demonstrated and all migration abnormalities detected were due to polymorphisms within the DRD2 gene. More recently, a correlation between DRD2 polymorphisms and cabergoline responsiveness was not found in lactotroph PitNETs [53]. Similarly, the link between DRD2 expression and treatment response to DA in somatotroph PitNETs is unclear. On the one hand, in vitro studies have shown correlation between DRD2 expression and GH response [54] that depend on the expressed DRD2 isoforms [55], but in vivo suppression of GH secretion by quinagolide in 24 somatotroph PitNETs did not correlate with DRD2 expression [48]. In addition, prolonged dopamine inhibition in humans caused by drugs, pituitary stalk dysfunction or direct hypothalamic damage did not induce lactotroph PitNETs or acromegaly. Altogether, these observations go against the presence of mutated D2DR or the loss of dopamine inhibition as primary causes of lactotroph or somatotroph tumors in humans, suggesting that the in vivo sensitivity to DAs might be affected by other mechanisms.

## 6. GHRH Receptor and Ghrelin Receptor 

GH pulsatile secretion is regulated by hypothalamic factors, GH-releasing hormone (GHRH), somatostatin and peripheral factors, including nutritional and metabolic signals [56]. Somatostatin, secreted by the delta cells of the pancreas, hyperglycemia, free fatty acids and IGF-1 itself, inhibits both GH and GHRH secretion. On the other hand, ghrelin (secreted in the stomach [57]), hypoglycemia, some amino acids, sleep, stress and exercise are physiological stimuli of GH secretion.

GHRH receptor (GHRH-R) is a class B of G-protein-coupled receptor [58]. GHRH is the native ligand of GHRH-R; its binding to the receptor in the somatotroph or mammosomatotroph cell leads to an increase in intracellular Ca^2+^ through cyclic adenosine monophosphate (cAMP)-dependent pathways and, as a result, GH secretion occurs (Figure 2). GHRH-R has been also linked to the proliferation of normal somatotrophs and may be involved in the growth of GH-secreting PitNETs via MAPK, among others [59,60]. Therefore, constitutive activation of GHRH-R might underlie certain GH-producing tumors.

In this regard, Pit-1 and GHRH-R mRNA expressions in silent somatotroph PitNETs and silent PRL PitNETs were similar to those found in the corresponding functioning PitNETs using a quantitative reverse transcriptase polymerase chain reaction method [61]. These results suggest that the cause of the absence of hormonal production and secretion in these tumors seems to not be in the receptor but in the downstream transcription signaling pathway leading to hormone secretion. Conversely, in vitro Pit-1 double negative human tumoral somatotroph and lactotroph cells, as well as murine mammosomatotroph cell line GH4C1 and in vivo GH4C1 subcutaneous xenografts, in nude mice have been shown to induce a decrease in cell proliferation and hormonal secretion [62]. Thus, gene therapy of Pit-1-derived tumors could be a promising target therapy in these tumors.

Likewise, somatic mutations of GHRH-R in the Gs α sub-unit (gsp oncogene) have been found in up to 40% of human GH-producing PitNETs, mainly in a densely granulated pattern [63], and have been shown to lead to constitutive activation of adenylyl cyclase and increased AMPc [64]. Increased intracellular cAMP levels lead to somatotrophic proliferation, hyperplasia and GH hypersecretion [65]. There are some clinical reports describing the transformation of a nonfunctioning PitNET to a clinically overt secretory tumor. Particularly, a patient with a DA-resistant PRL-secreting PitNET which evolved into acromegaly has been described, involving an increase in somatotroph cell number in the tumor and the expression of the gsp oncogene [66]. These results further support the notion that the gsp oncogene is a mutational change associated with somatotroph growth and transformation.

Further, gsp mutations in human GH-secreting PitNETs may upregulate the expression of the GH secretagogue receptor (GHSR), a receptor that enhances GH pulsatility and amplitude [67] as well as PRL secretion [68]. Moreover, the expression of GHSR was positively correlated with tumor size and invasiveness in patients with acromegaly [69]. Nevertheless, the relationship between gsp mutation and somatotroph PitNETs is still controversial and not fully understood. In the study by Larkin et al. [70], the granulation pattern, but not gsp or GH-R mutation, was associated with clinical characteristics in somatostatin-naive patients with somatotroph PitNETs.

## 7. Somatostatin Receptors

In contrast to GHRH and GHSR action, somatostatin inhibits both GH and PRL secretion throughout a somatostatin receptor (SST-R), which is another G-protein-coupled receptor. There are five subtypes of SST-R (SST-R1 to SST-R5), variably expressed in both normal and tumor tissues. The main subtypes expressed in somatotroph tumors are SST-R2 and SST-R5 in variable ratios and, to a lower extent, SST-R3, SST-R1 and SST-R4 [71,72]. Studies evaluating the resistance of these tumors to first-generation somatostatin receptor ligand (fg-SRL) are quite concordant in high SST-R2 expression-associated favorable response, while negative immunohistochemistry used to be found in unresponsive tumors [73,74,75]; furthermore, patients with a good response to octreotide have a SST-R2/SST-R5 ratio ≥1.3 [71,72]. However, the determinants of responsiveness to fg-SRLs in non-selected somatotropinomas are related to the combined differential expression of different biomarkers, SSTRs being one of them, along with E-cadherin, Ki-67, beta-arrestines and others being involved in final single-case responsiveness [76].

In contrast, studies evaluating the response to pasireotide, a second-generation SRL with multiligand properties [77,78], have shown discordant results. Some studies have shown that high SST-R5 immunoreactivity might predict a good response to pasireotide [74,79] and low SST-R5 a poor response [80]. On the contrary, other studies suggested that the effects of pasireotide on somatotroph tumors are in fact also driven by SST2 [81,82,83].

The immunohistochemical analysis of SST-R in PRL-secreting PitNETs demonstrated that SST-R5 was the most frequent, followed by SST-R2 and SST-R1 [84]. In agreement with that, some groups have reported beneficial effects of pasireotide in resistant PRL-secreting PitNETs on both tumor shrinkage and PRL levels [85,86,87]. Likewise, SST-R1 associated to SST-R2 and SST-R5 was present in mixed GH–PRL-secreting tumors and the selective in vitro activation of SST-R1 in these mixed tumors led to a significant reduction in both hormone secretion and cell viability [88].

However, the observed inhibitory effect of SRL is not always explained on the basis of binding affinities, suggesting that a ligand-induced dimerization process between receptors or that a combination of differentiation post-receptorial pathways may occur. In fact, recent evidence has shown homo- and heterodimers, such as SSTR5/DR2 or SST-R5/SST-R1, with enhanced functional activity [89]. In this regard, SST-R1 seems to remain monomeric after ligand activation and SST-R1-selective agonist treatment affects both hormone secretion and cell survival. Therefore, SRLs that bind selectively to SST-R1 with high affinity or together with other SST-R subtypes could be a new therapeutic option.

However, the results regarding the in vivo expression of SST-R in pituitary tumors vary among studies due to the use of quantitative PCR or immunohistochemistry with polyclonal or monoclonal antibodies; for this reason, among others, the assessment SST-Rs has not yet been implemented routinely in the clinical practice.

Potassium channel subunit–encoding gene KCNAB2 is highly differentially methylated between secretory and non-secretory PitNETs, with greater KCNAB2 methylation being detected in the former. KCNAB2 negatively regulates members of the voltage-gated potassium channel (Kc). Modulating the expression of Kc in GH3 cells lines resulted in concordant changes in both the expression of GH mRNA and downstream secretion of GH. Moreover, the Kc modulatory drug quinidine negatively regulates both GH and PRL secretion in a dose-dependent manner [90]. All these findings suggest KCNAB2 as a potential new target and pharmacological candidate to be considered in the development of clinical therapeutics for acromegaly, particularly in mammosomatotroph tumors.

## 8. Classification of GH–PRL-Secreting PitNETs

**Figure 3:**
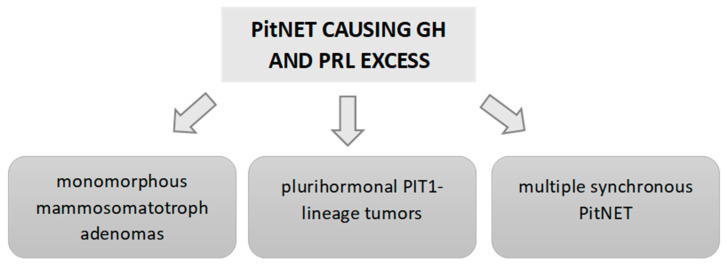
Causes of GH and PRL hypersecretion due to a PitNET.

As we stated above, according to the latest classification of PitNETs of the World Health Organization (2022) [3], GH/PRL co-secreting tumors include dimorphous PitNETs composed of GH- and PRL-secreting cells (mixed somatotroph–lactotroph tumors), as well as the monomorphous PitNETs (producing both PRL and GH within the same cell) [3]. Mammosomatotroph tumors are then Pit-1 monomorphous tumors and express ERα and PRL in many cells. They are generally composed of densely granulated somatotrophs and sparsely granulated lactotrophs, but sometimes they are composed of sparsely granulated somatotrophs and sparsely granulated lactotrophs. Under electron microscopy, they also resemble somatotrophs, but have more variable sizes and shapes of secretory granules that vary from 200–2000 nm and may show the misplaced exocytosis that is typical of lactotrophs [91] (Figure 3).

This latest PitNET WHO classification [3] also includes a new subtype of PitNET responsible for the secretion of TSH, GH and PRL: the mature plurihormonal PIT-1-lineage tumor, which is composed of monomorphous cells. These tumors resemble a mammosomatotroph tumor, secreting GH and PRL, but also express variable GATA and βTSH, producing overt acromegaly associated with hyperprolactinemia. This immature subtype can also develop hyperthyroidism [4] (Figure 3).

On the contrary, mixed somatotroph–lactotroph tumors are composed of two distinct cell populations: somatotrophs and lactotrophs. For this PitNET subtype, the tumor express Pit-1 in all tumor cells, but only the cells that express PRL also express Erα [21].

Another point highlighted in the latest classification is the importance of distinguishing between multiple synchronous PitNETs and mixed tumors [92,93]. The former are double or multiple PitNETs of the pituitary, composed of two or more distinct tumors co-located in the gland. Multiple synchronous PitNETs represent less than 1.5% of PitNETs. For example, in the German Registry of Pituitary Tumors [94] that includes a total of 16,283 PitNETs, only 1.4% (232 cases) had more than 1 PitNET. Of these cases, 38 were double PitNETs, 2 were triple PitNETs and the remaining PitNETs associated with other sellar neoplasms or tumor-like lesions.

## 9. Clinical Aspects and Outcomes of GH–PRL PitNETs 

### 9.1. Prevalence and Epidemiological Aspects

The percentage of GH-secreting PitNETs that co-secrete prolactin varies across studies and depends on factors like the diagnostic criteria used. In general, co-secretion is estimated to be present in about 24–30% [4,95]. However, the coexistence of hyperprolactinemia in patients with acromegaly reaches a prevalence of up to 40% in some series and predicts worse surgical outcomes [96]. On the other hand, among 94 patients with GH-secreting PitNETs, 56% were pure GH-secreting, 30% mammosomatotrophs and 14% mixed somatotroph–lactotroph PitNETs, as assessed by immunohistochemistry in a recently published series [4]. This figure is in agreement with other less recent studies [97,98]. It is possible that the higher reported prevalence in most recent studies is related to the advances in PitNET characterization [3]. Nevertheless, in most studies, this heterogeneity lies in the classification of GH–PRL PitNETs used regarding hormonal and/or immunohistochemical data, and, as far as our knowledge reaches, no previous studies have differentiated GH–PRL PitNETs and GH PitNETs based on transcription factor results (Table 2).

Another important point is the possibility of developing acromegaly in patients with a known PRL-secreting PitNET. In this regard, Andersen [99] reported that 3 cases with PRL-secreting PitNETs out of 78 total patients developed a clinical and biochemical acromegaly after a mean follow-up of 43 months. These patients had a normal or low GH level and/or a normal IGF1 level at first diagnosis. Considering these results, they proposed that annual IGF1 measurement should be carried out as a screening test in patients with PRL-secreting PitNETs. They also emphasized that the diagnosis of GH–PRL co-secretion may be underdiagnosed in patients with PRL-secreting PitNETs since it is known that dopamine-D2 agonist decreases IGF1 levels in patients with acromegaly. On the contrary, other authors suggested that IGF1 levels may increase paradoxically with DA treatment for PRL-secreting PitNETs [100,101]. In the study by Akirov et al. [100], the mean IGF1 increase while undergoing cabergoline treatment was 1.7 ± 0.4 × the upper limit of normal. Other previous studies described similar results, reporting an increase of serum GH/IGF1 levels with DA [102,103].

Regarding gender, most studies did not find differences across the different subtypes (pure GH-secreting PitNETs, mammosomatotroph and mixed somatotroph–lactotroph PitNETs) [4], nor between acromegaly patients with and without associated hyperprolactinemia [104,105]. Studies comparing GH–PRL-positive immunostaining tumors and only GH positive tumors could not detect differences in age and gender presentation between both groups [95]. Thus, as is observed in acromegaly in general, females are slightly more affected than men (around 55–60% of the cases are women) [106]. Nonetheless, one large study including 529 patients with acromegaly described a higher rate of females among patients with acromegaly and hyperprolactinemia than among those acromegalic patients with normal PRL levels (64.7 vs. 50%, *p* = 0.001) [96]. Differences across studies may be justified by the different classifications employed in each study (e.g., PRL levels, different PRL thresholds, immunohistochemistry) (Table 2).

Some studies have described that mammosomatotroph PitNETs are more common in young patients with gigantism [107]. Along this same line, studies comparing acromegaly patients with and without hyperprolactinemia described an age 3 to 5 years younger in the group of GH–PRL PitNETs compared with GH PitNETs [96,104]. In fact, while densely granulated somatotroph tumors has been found as the most frequent cause of acromegaly in adults, mammosomatotroph tumors producing GH and PRL have been reported as the most common tumors in young patients with acromegaly and in cases of childhood-onset gigantism [108]. Moreover, one study comparing patients with GH–PRL PitNETs and PRL PitNETs also detected a younger age at diagnosis in the former (38.13 ± 13.31 vs. 41.95 ± 14.70 years; *p* = 0.025) [104] (Table 1). Nevertheless, more recent studies did not detect differences in age presentation of the acromegaly across the different GH-secreting PitNETs subtypes [4]; neither were differences found when comparing patients with positive staining only for GH and with positive staining for both PRL and GH [95] nor when comparing patients with normoprolactinemia and patients with high PRL levels [105]. The median age of patients with PitNETs causing acromegaly is between 42 and 46 years, independently of the anatomopathological subtype, according to Liang [4] (Table 2). 

**Table 2:** Epidemiological and clinical data of GH–PRL-secreting PitNETs and GH PitNETs.

	Number of Cases	Definitions	Gender and Age	Tumors Size and Invasiveness	Hormonal Data	Surgical and Medical Outcomes
PATHOLOGICAL CLASSIFICATION (based on PRL and GH staining)						
Varlamov, 2020 [109]	Bihormonal group (9 MSA,10 MSLA), 30 DGA, and 28 SGA	Based on 2017 WHO classification staining for GH, PRL and cytokeratin	Patients in the bihormonal group were older than SGA. No differences in sex	Bihormonal adenomas did not differ in tumor size from DGA or SGA and were less frequently invasive than SGA	Bihormonal adenomas had higher baseline IGF1 index compared to SGA	No difference in the surgical remission rates across the groups
Liang Lv, 2019 [4]	53 PSA, 28 MSA and 13 MSLA	Based on staining for GH and PRL *	No differences in sex and age across subtypes	MSLAs were larger and more invasive than MSA and PSA	No differences in baseline GH and IGF-1 level nor in hypopituitarism prevalence across subtypes	MSLAs had worse long-term biological remission rate than MSAs and PSAs
Rick, 2019 [95]	69 GH-positive staining tumors and 22 with GH–PRL-positive staining	Based on staining for GH and PRL	No differences in sex and age between groups	No differences in tumor size nor in cavernous sinus invasion	GH–PRL tumors had higher serum PRL and IGF1 levels than GH tumors	Patients with GH–PRL tumors were less likely to achieve remission with surgery than GH tumors
HORMONAL CLASSIFICATION (based on serum PRL levels)						
Wang, 2012 [104]	182 patients with normal PRL levels and 97 with high levels of PRL	PRL threshold: any PRL elevation above the ULN	The GH group had a higher age than GH–PRL group. No differences by gender	The GH group had a smaller mean maximal diameter	Higher GH levels in the GH group than in the GH–PRL group	No differences in surgical control rates were observed between both groups
Laethem, 2020 [105]	25 with normal PRL levels and 19 with high levels	PRL threshold: any PRL elevation above the ULN	No differences in age at diagnosis and gender between both groups	No differences in tumor size and invasiveness between groups	Similar GH and IGF1 levels between groups	The rate of surgical remission of acromegaly was not reported
Guo, 2022 [96]	322 with normal PRL levels and 207 with high PRL levels	Hyperprolactinemia and hypopituitarism in acromegaly and effect of pituitary surgery: long-term follow-up on 529 patients	Patients with hyperprolactinemia were younger and more likely to be females	Patients with hyperprolactinemia had larger and more invasive tumors	Higher baseline and GH nadir levels in patients with hyperprolactinemia	Patients with preoperative hyperprolactinemia had higher recurrence rates after surgery than those without
IN VITRO PRESENCE OF DETECTABLE PRL SECRETION						
Gatto, 2022 [110]	15 with normal PRL and 8 with high PRL	Based on in vitro secretion of GH and/or PRL **	No differences in age and gender between both groups	The prevalence of macroadenomas was comparable	No differences in GH and IGF1 levels between groups	No differences in the response to octreotide and cabergoline between groups

DGA: densely granulated GH adenomas; SGA: sparsely granulated GH adenomas; PSA: pure somatotroph adenoma; MSA: mammosomatotroph PitNETs; MSLA: mixed somatotroph–lactotroph adenoma; ULN: upper limit of normality. * MSA was positive staining for GH and PRL in the same cells; MSLA was composed of a dual-cell population that was respectively positive for GH and PRL; PSA was positive only for GH; ** Tumors were classified as mixed tumors in those samples in which both GH and PRL secretions were detectable in the conditioned medium, while the pure somatotroph tumors showed GH secretion alone.

### 9.2. Clinical and Hormonal Behavior 

The clinical behavior is mostly determined by the pathological subtype of PitNET causing the GH and PRL excess, since it has been reported that plurihormonal Pit-1-lineage tumors tend to be more invasive and, therefore, have a poorer prognosis than GH-secreting PitNETs [20]. In addition, patients with GH–PRL PitNET, as could be expected, have additional hyperprolactinemia-induced symptoms, such as decreased libido and menstrual cycle alterations. Patients with GH–PRL PitNETs are usually more symptomatic, including a higher prevalence of coarse facial features, polyuria and polydipsia, large hands and feet and diabetes mellitus, than GH PitNETs, probably because they show higher levels of IGF1 and PRL [101]. In patients with plurihormomal Pit-1-lineage tumors, the clinical presentation is almost identical to mammosomatotrophs PitNETs, but the patients may also have hyperthyroidism symptoms [107]. 

Regarding the hormonal parameters in patients presenting GH–PRL co-secretion, it has been described that dual staining PitNETs (positive for GH and PRL) presented significantly higher serum IGF1 levels than isolated positive GH PitNETs [95]. In this same line, other authors described higher levels of GH (*p* = 0.004) and GH nadir after glucose tolerance test (*p* = 0.003) [96]. However, no differences in baseline IGF1 nor baseline GH levels among patients with pure GH-secreting PitNETs, mammosomatotrophs and mixed somatotroph–lactotroph PitNETs were described in other series [4]. Nevertheless, some series found a higher baseline GH level in patients without associated hyperprolactinemia than in those without it (42.4 ± 30.5 ng/mL vs. 23.4 ± 15.8, *p* < 0.001) [104]. The real explanation of these differences across studies in not fully understood; altogether, the expected results are to detect higher IGF1 and GH levels in GH–PRL PitNETs than in GH PitNETs since most studies describe a larger tumor size in the former [4,95,104], and it is known that, in general, there is a positive correlation between IGF1 levels and tumor size in acromegaly.

The reported levels of PRL widely vary in patients with GH–PRL PitNETs across the different series. PRL levels are usually higher than 41.5 ng/dL in these tumors, although only 23% of mixed somatotroph–lactotroph PitNETs have a clear high level of PRL, which is usually found in standard lactotroph PitNET cases (>200 ng/mL)) [4]. However, it is important to note that a significant percentage of cases with double immunostaining have normal PRL levels, as exemplified in the Rick series [95], were 27.3% of dual-staining tumor patients did not have PRL elevation and, among the hyperprolactinemic patients, 22.7% had PRL elevations below 41.5 ng/dL.

Regarding the prevalence of preoperative hypopituitarism, despite the larger tumor size of GH–PRL-secreting PitNETs described in some series [4,95,104], no significant differences in the rates of hypoadrenalism, hypothyroidism and hypogonadism have been detected across the different subtypes in some series [3]; other authors did not describe this aspect [95,104].

### 9.3. Radiological Features

Mixed somatotroph–lactotroph PitNETs are usually larger than mammosomatotroph PitNETs [4] (Table 2). In the same line, it has been described that GH tumors causing hyperprolactinemia were larger than those without PRL increase [96,104]. In accordance, other series found that the proportion of preoperative macroadenomas was significantly higher in patients with hyperprolactinemia than without it (60.4% vs. 45.1%, *p* = 0.041) [104]. 

Cavernous sinus invasion has been reported to be more common in mixed somatotroph–lactotroph PitNETs than in mammosomatotroph PitNETs [4]. Accordingly, cavernous sinus invasion is also more frequent in GH PitNETs with associated hyperprolactinemia [96]. In the same line, Liang et al. [4] reported that the frequency of cavernous sinus invasion differed by subtype: 32.1% of pure GH PitNETs, 7.1% of mammosomatotroph and 7.7% of mixed tumors for left cavernous sinus invasion (*p* = 0.0145) and 7.5% of pure GH, 0.0% of mammosomatotroph and 38.5% of mixed tumors for right cavernous sinus invasion (*p* = 0.0003). Nonetheless, other studies comparing patients with PitNETs with positive staining only for GH and those with positive GH and PRL staining were not able to demonstrate differences in tumor size and the rates of cavernous sinus invasion between both groups, despite the greater degree of IGF1 elevation in patients with GH–PRL PitNETs [95]. Conversely, some authors [109] found that PitNETs secreting GH and PRL were less frequently invasive (15.8% vs. 57.1%, *p* = 0.005) than PitNETs that only secreted GH, despite higher IGF1 indexes (3.3 vs. 2.3, *p* = 0.040) in the former [109]. Differences across studies would be explained by the different proportions of densely and sparsely granulated somatotroph tumors that have been included in each study in the control group (GH-secreting PitNETs). For example, in the Varlamov study, 50% of the patients in the control group had sparsely granulated GH PitNETs [109], whereas, in the Liang study [4], 66% of the tumors were sparsely granulated. This information is not available in other series [95]. 

Another interesting finding of the study of Varlamov et al. [109] was that GH- and PRL-secreting PitNETs more frequently had a cystic component on MRI than sparsely granulated GH PitNETs and densely granulated GH PitNETs (52.6% vs. 14.3% and 22%, *p* = 0.005 and 0.033, respectively). In addition, as an important point, they detected that cystic tumors in patients with acromegaly had lower rates of biochemical remission after surgery, regardless of histological subtype.

Because mammosomatotroph PitNETs are usually densely granulated tumors, they generally have low intensity on T2-weighted MRI images [20]. However, this finding has not been evaluated in other series [4,95,96,104,105,110] and other authors found a similar proportion of hypointensity, isointensity and hyperintensity in T2-MRI sequence in GH–PRL-secreting PitNETs [109]. These differences may be justified by the proportion of densely granulated tumors, which varies across studies. For example, some series found a greater proportion of sparsely granulated tumors (57%) than densely granulated tumors in mammosomatotroph PitNETs, similar to those described in pure GH-secreting PitNETs (66%), but higher than in the mixed group (38.5%) [4]. Nevertheless, it should be taken into account that there are other factors that may determine MRI T2 intensity such as collagen content, fibrosis and amyloid deposition [111].

### 9.4. Surgical and Medical Outcomes

It has been reported that mixed somatotroph–lactotroph PitNETs had worse long-term biological remission rate than mammosomatotrophs and GH-secreting PitNETs (15.4% vs. 50.0% and 26.4%, respectively; *p* = 0.037) [4]. Consistent with this, Wang et al. [104] found that GH PitNETs with associated hyperprolactinemia often showed larger lesions despite lower preoperative GH levels than GH-PitNETs without associated hyperprolactinemia. The worst surgical outcomes may be related to the larger tumor size in mixed somatotroph–lactotroph PitNETs compared to pure GH-secreting PitNETs [4]. Other authors have also described a poorer response to medical therapy and a higher risk for recurrence in tumors with positive staining for GH and PRL than in single-staining PitNETs despite similar tumor size [95].

Supporting the finding of worse outcomes in GH–PRL PitNETs, Rick et al. [95] reported that double-staining tumor patients were significantly less likely to experience postoperative biochemical remission than single-staining (20.0% vs. 67.9%, *p* = 0.01) and also had a higher risk of recurrence (18.2% vs. 7%). In fact, single-staining tumors were significantly associated with remission after pituitary surgery (odds ratio [OR] = 7.0, *p* = 0.02). In addition, they observed that patients with dual-staining tumors required a higher mean postoperative dose of pegvisomant, cabergoline and lanreotide than single-immunostaining tumors. Similarly, a study on 529 patients with acromegaly found that patients with preoperative hyperprolactinemia had higher recurrence rates than those without hyperprolactinemia [hazard ratio (HR) = 1.39 (1.08–1.79); *p* = 0.012] [96]. In addition, the median recurrence time after surgery was shorter in patients with hyperprolactinemia than in those with normal PRL levels [96]. Despite these findings, other studies have detected a similar rate of recurrence when comparing acromegaly patients with and without associated hyperprolactinemia (7.1 vs. 11.3%, *p* = 0.185) [104], although it should be noted that the rate of surgical remission was also lower in GH–PRL PitNETs that in GH-PitNETs in this series (69.1% vs. 80.7%, *p* = 0.037) (Table 2).

It could be expected that those patients with associated hyperprolactinemia had a higher response rate to DA due to a higher expression of DRD2 in patients with hyperprolactinemia than in those without. Nevertheless, a meta-analysis focused on the response to cabergoline in acromegaly [112] showed that this response is dependent on the IGF1 baseline levels, with greater chances to achieve IGF-1 level normalization with lower basal IGF-1 levels, regardless of the presence or absence of hyperprolactinemia. Along this same line, a recent in vitro study has described a head-to-head comparison between octreotide and cabergoline in inhibiting GH secretion in primary cultures of GH- and GH-/PRL-secreting PitNETs. As main findings, they observed that octreotide showed a slightly higher efficacy compared with cabergoline (GH decrease −39.5% vs. −32.5%, *p* = 0.079), and the effect of both drugs was superimposable in GH/PRL co-secreting tumors (−42.1% vs. −44.8%) [110]. Notably, in this study, DRD2 and SST1 mRNA levels were significantly higher in GH-/PRL-secreting tumors than in pure GH-secreting ones. However, a higher efficacy of DA in patients with hyperprolactinemia than without was reported in a few studies [113].

## 10. Future Directions and Conclusions

Understanding the molecular mechanisms in tumorigenesis is relevant. We have reviewed potential mechanisms involved in mixed and pluri-hormonal Pit-1 tumorogenesis. These mechanisms may represent potential targets for pharmacological treatment or have an impact on the prediction of tumor recurrence. The new PitNET classification has made an important and significant change in the fundamental concept of pituitary tumor understanding, enriching our knowledge of these special tumors in a way that will impact our future approach and clinical practice. New studies updating the samples with transcription factor studies are warranted. Currently, there are several concerns about the clinical and hormonal characteristics of GH/PRL co-secreting PitNETs since most of the studies focused on these types of tumors are retrospective and have included a limited number of cases. In addition, the definition of GH/PRL PitNET is widely variable across these studies. In fact, most of them based the definition of GH/PRL co-secreting PitNETs on serum prolactin levels and not on the results of the transcription factors (Table 2). Moreover, several series did not report PRL and GH immunohistochemistry results for the classification of these tumors. Thus, the first step for advancing the knowledge of patients with GH/PRL PitNETs is to reach a consensus on the definition of GH/PRL PitNETs; in this regard, the latest WHO pituitary tumor classification [3] seems to be the most appropriate way. Another point to consider is that few studies have compared the surgical and medical outcomes of patients with GH/PRL PitNETs to those with GH PitNETs. It is important to characterize the different clinical and biochemical profiles of these patients, since the identification of treatments with a greater effect on tumor size and on biochemical control in patients with GH/PRL PitNETs (or the ones that will be resistant to medical therapy) would allow us to implement a personalized approach, leading to earlier and better control of the disease. We consider that the ideal approach to obtain more information about GH/PRL tumors would be carrying out multicentric studies that follow the latest WHO classification recommendations for tumor classification, combining molecular and clinical information to determine the outcomes of these patients.

The composite structure of the adult pituitary has a dual embryonic origin. The posterior lobe consists of nervous tissue arising from the diencephalon and represents an extension of the hypothalamus and the anterior lobe derives from the oral ectoderm. 

The dorsal and ventral side of the embryonic pituitary generate proliferative and positional signals, which regulate the expression of transcription factors. On the ventral side, when SF1, GATA and ERα are activated, they determine the gonadotroph linage. T-Pit signal, which differentiates the most dorsal cells into corticotroph (C) (in orange) and Pit-1 induced in the caudomedial region of the pituitary gland, is the most complex, with five monomorph cell types: somatotroph (S) (in yellow), lactotroph (L) (in green), thyrotroph cells (T) (in violet), mammosomatotrophs (in yellow mixed texture) and plurihormonal linage tumor (in violet mixed texture).

The autocrine-released GH and PRL bind to prolactin or growth hormone receptor via Janus kinase-2-signal transducer and activation of transcription-5 (JAK–STAT5), (PI3K-Akt-mTOR) or the MAPK pathways, which mediates changes in transcription and differentiation, preventing hormone formation and cell proliferation (negative short loop).

Growth hormone-releasing hormone receptor (GHRHR) activation results in the secretion and production of growth hormone through cyclic adenosine monophosphate (cAMP)-dependent pathways. GHRHR activation induces adenylyl cyclase (AC), which converts ATP to cAMP, stimulating protein kinase A (PKA) regulatory subunits which increase intracellular Ca^2+^ via voltage-gated Ca^2+^ currents, thus favoring growth hormone secretion. GHRHR also activates MAPK pathways; thus, it is related to cellular growth. 

The downstream signals of the growth hormone secretagogue receptor includes, among others, the phospholipase C (PLC)–inositol triphosphates (IP3) pathways. IP3 is soluble and diffuses through the cell, where it binds to its receptor, which is a calcium channel located in the endoplasmic reticulum. When IP3 binds to its receptor, calcium is released into the cytosol, thereby activating various calcium regulated intracellular signals, potentiating both GH and prolactin secretion.

After dopamine binding to dopamine receptor type 2, K^+^ channels are activated, leading to reduced calcium influx, which results in an immediate suppression of prolactin and/or GH release. Further decreases in intracellular calcium are achieved by inhibition of PLC and PKC. The main mechanism for the suppression of PRL or GH gene expression is through AC activity inactivation, resulting in the suppression of PRL or GH gene expression, cell proliferation and cell size. D2 via G0 also activates phosphatidylinositol 3-kinase (PI3K), and mitogen-activated protein kinase (MAPK) pathways to prevent cell proliferation. Similar to D2R, somatostatin receptor (SSTR) inhibits the secretion/synthesis of GH/ PRL, mainly through AC inhibition, lowering AMPc levels and decreasing intracellular Ca^2+^ concentration via activation of K^+^ channels and the inhibition of voltage-dependent Ca^2+^ channels. The anti-proliferative effects of SSTR are mediated, among others, via PI3K-/Akt-impairing cell proliferation.

## Figures and Tables

**Table 1:** Search terms used for the preparation of the review.

Search Terms	Total of Results
pituitary [TI] AND ontogeny [TI]	119
mammosomatotroph [TIAB]	90
molecular [TIAB] AND acromegaly [TI]	87
molecular [TIAB] AND prolactinoma [TIAB]	72
prolactin receptor [TI] AND molecular [TIAB]	102
growth hormone receptor [TI] AND molecular [TIAB]	161
somatostatin receptor [TI] AND molecular [TIAB] AND pituitary [TIAB]	47
dopamine receptor [TI] AND molecular [TIAB] AND pituitary [TIAB]	18
GHRH receptor [TI]	71
Ghrelin receptor [TI] AND pituitary [TI]	36
plurihormonal [TIAB] AND pituitary [TI]	189
GH [TI] AND prolactin pituitary [TI]	3
mixed pituitary adenomas [TI]	4
acromegaly [TI] AND plurihormonal [TI]	5

TI: title; TIAB: title or abstract.
